# Supplementary material for: The Impact of Visual Cues, Reward, and Motor Feedback on the Representation of Behaviorally Relevant Spatial Locations in Primary Visual Cortex
Source: Cell Rep. 2018 Sep 4;24(10):2521–8. doi: 10.1016/j.celrep.2018.08.010 (PMC6137817; doi:10.1016/j.celrep.2018.08.010)
Supplement: Document S1. Supplemental Experimental Procedures and Figures S1 and S2 [file mmc1.pdf]

Cell Reports, Volume *24*

## Supplemental Information

# The Impact of Visual Cues, Reward, and Motor Feedback on the Representation of Behaviorally Relevant Spatial Locations in Primary Visual Cortex

**Janelle M.P. Pakan, Stephen P. Currie, Lukas Fischer, and Nathalie L. Rochefort**

## SUPPLEMENTAL FIGURES

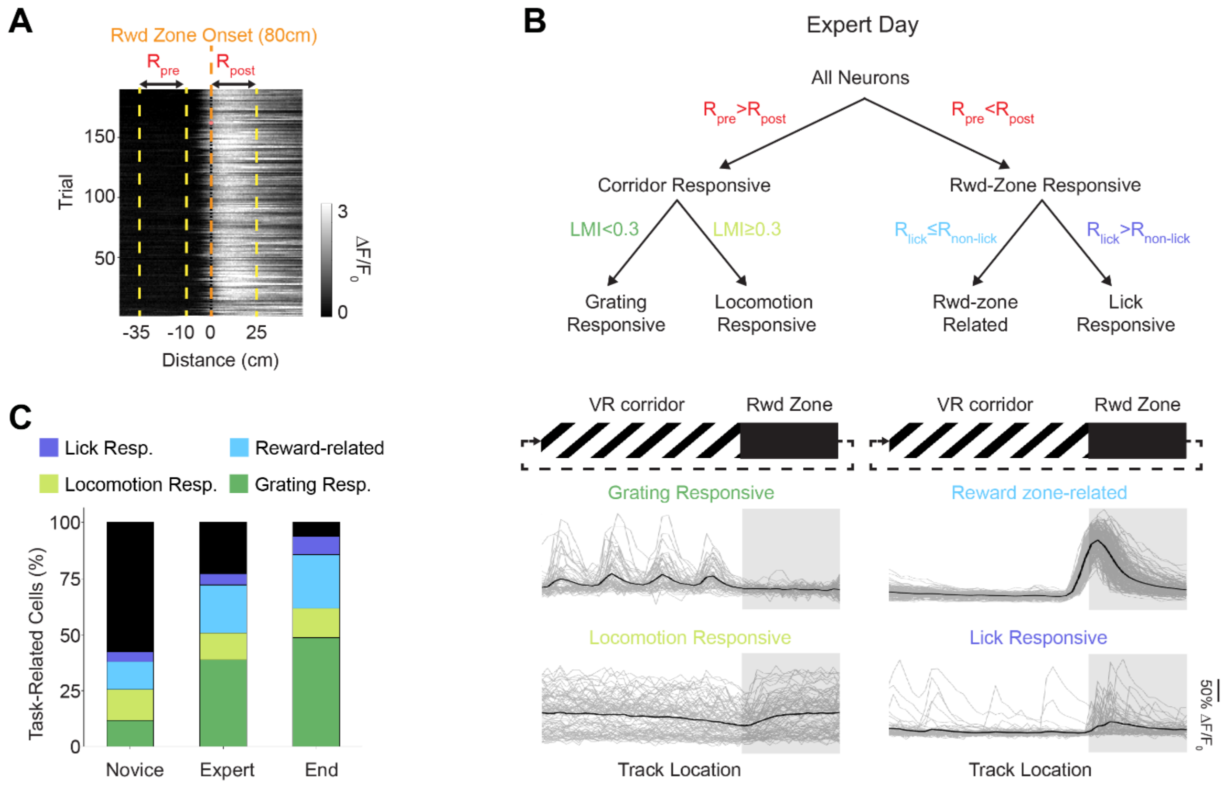


**Figure S1. Response profile of V1 neurons during learning of a visually-guided reward task. Related to Figure 2.**

(A) Normalised $\Delta F/F_0$ around the reward zone onset (orange dashed line) for a neuron defined as task-related ($R_{pre}$ vs $R_{post}$; $p<0.001$, Wilcoxon signed rank test). The 25 cm block used to define activity before ($R_{pre}$) and after ($R_{post}$) the reward zone onset are marked for reference (yellow dashes lines).

(B) Flow chart defining the characterisation of different response types within the task-responsive V1 population. All task-responsive neurons were first separated into those significantly decreasing (corridor responsive; $R_{pre} > R_{post}$; $p<0.001$, Wilcoxon signed rank test) or increasing (reward zone responsive; $R_{pre} < R_{post}$; $p<0.001$, Wilcoxon signed rank test) their activity at the reward-zone onset. The corridor responsive neurons were sub-divided based on whether their locomotion modulation index (LMI) was < 0.3 (grating responsive) or > 0.3 (locomotion responsive). The reward-zone responsive neurons were sub-divided based on whether their activity during licking ($R_{lick}$) was significantly higher than their activity during an equal number of non-licking ($R_{non\text{-}lick}$) periods along the virtual corridor (lick responsive; $R_{lick}$ vs $R_{non\text{-}lick}$; $p<0.001$, Wilcoxon signed rank test) or not (reward-zone related). Lower panels show $\Delta F/F_0$ for single trials (grey lines) and average response (black line) for representative neurons classified in each of the response types.

(C) Bar chart showing the evolution of the percentage of the different neuronal response types during learning. At each stage (novice, expert and end-point days) neurons were classified as described in B.

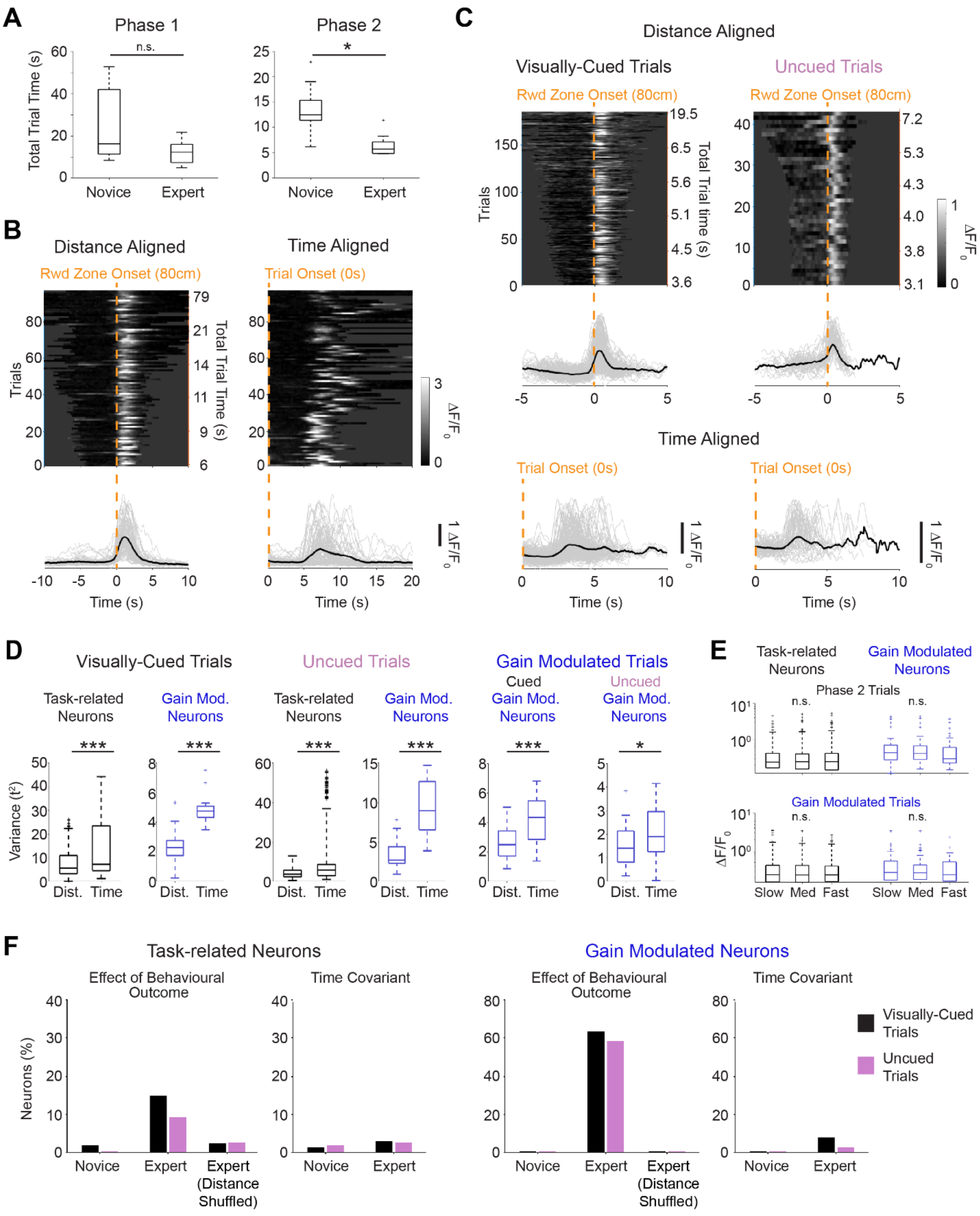


**Figure S2. Contribution of trial time to task-related and gain-modulated neuronal responses. Related to Figure 2 and Figure 3.**

(A) The average trial time per animal during novice and expert days is shown for both learning phases (Phase 1: p=0.219; Phase 2: p=0.016; n=7; Kruskal–Wallis test), showing that during the expert day animals completed trials faster and at a less variable rate. Boxplots represent median, 1st and 3rd quartile, and 1.5 interquartile range (IQR).

(B) The responses of an example neuron are shown (same neuron as shown in Figure S2B) for the expert day (Phase 1), over time and for each trial. Responses are aligned either to the reward-zone onset (at 80 cm; left panel) or to the start time of each trial (trial onset, 0 s; right). In the left panel, the trials are sorted by total trial time (indicated on the right y-axis). The bottom panels show the traces ($\Delta F/F_0$) of each trial (grey lines) and the mean across trials (black line). Note that this neuron responds reliably at the reward-zone onset (80 cm) for the fastest as well as slowest trials, which can differ by more than an order of magnitude (range 6-79 s). When responses are aligned to trial onset, the peak responses are more variable across trials (right panel).

(C) Example responses are shown from expert day for Phase 2 for visually-cued (left) and uncued (right) trials aligned to distance onset (top two panels; reward-zone onset, 80 cm) and trial times are sorted from fastest to slowest (indicated on the right y-axis). Note that even for uncued trials, there is a reliable response at the reward-zone onset (80 cm), regardless of whether the animal completes a trial quickly (3.1 s shortest trial time) or takes more than double that time (7.2 s). This indicates that the response to the uncued reward-location is not an entrainment response across trials of the same length but is specific to a spatially-defined reward location. In the bottom panels, $\Delta F/F_0$ traces for each trial (grey lines) and the mean across trials (black line) are shown aligned to the trial onset and show more variability.

(D) Trial-by-trial variability is quantified for all task-related neurons (black; see also Figure 2) and all gain-modulated neurons (blue; see also Figure 3E-F) on expert day Phase 2, by taking the variance of the time at peak response for each trial when trials were aligned either by distance (Dist.; reward-zone onset, 80 cm) or by trial start (Start; trial onset, 0 s). In both populations of neurons and for both visually-cued trials and uncued trials, as well as gain modulated trials (cued and uncued) we found significantly less variability when responses were aligned to distance onset (***$p<0.001$, *$p=0.0315$; task-related neurons $n = 613$; gain modulated neurons $n = 38$; Kruskal–Wallis test). Boxplots represent median, 1st and 3rd quartile, and 1.5 IQR.

(E) The mean $\Delta F/F_0$ surrounding the reward zone (10 cm before and after reward zone onset) is shown for the slowest trials (slow; 1st quartile), fastest trials (fast; 3rd quartile), and all trials in between (med) for the task-related (black) and gain modulated (blue) population of neurons. The top panel shows data from Phase 2 (non-gain modulated trials) on expert day and the bottom panel shows data from gain modulated trials (Phase 3). We found no significant difference between any condition ($p>0.050$ for all conditions, task-related neurons $n=613$; gain modulated neurons $n=38$; Kruskal–Wallis test). Boxplots represent median, 1st and 3rd quartile, and 1.5 IQR.

(F) The percentage of task-related (left) and gain modulated (right) neurons in V1 with significantly different responses according to behavioural outcome (successful vs missed trials) according to a multivariate analysis of covariance (MANCOVA) and taking into account total trial time as a covariate, for each of the different stimulus conditions (visually-cued [black] vs uncued [purple] trials). On expert days, the proportion of neurons showing responses affected by behavioural outcome was larger than on novice days (especially in gain modulated neurons). When the distance along the track was shuffled (so that random lengths of the track outside the reward-zone were sampled; distance shuffled), the effect of behavioural outcome was abolished. Note that time was not a significant covariate for the majority of neurons on either novice or expert days for either stimulus condition. For all conditions significance was determined by $p<0.001$.

## SUPPLEMENTAL METHODS

*Water deprivation and training schedule*

Mice were put on a 1 ml/day water restriction regime to increase motivation during behavioral training. This regime maintained bodyweight at 85-90% of their free feeding weight, calculated as the mean of the last 3 days before water restriction. Where possible, mice were trained at the same time every day and generally in the first few hours of their dark phase. Training consisted of a 30 minute session, 5 days per week, followed by a 2-day break. Successful trials were rewarded with a droplet of water (~ 5µl/reward). Following each session, the volume of water consumed during the task was supplemented to 1ml if necessary.

*Virtual Reality gain modulation*

For phase 3 the gain relating the rotation of the cylindrical treadmill to the progression in the virtual corridor was reduced from 1 to 0.75. This resulted in a mismatch between the movement on the treadmill and movement through the virtual environment. On these trials, the same number of steps took animals only 75% of the distance along the virtual corridor compared to a normal trial (see Figure 3E). In these trials, the reward was given at 80 cm along the virtual corridor (in virtual space), which corresponds to 107 cm in physical distance travelled. Therefore, the reward was actually given after the expected reward onset based on physical distance along the treadmill (the expected location being at 80 cm of physical distance travelled and correspondingly 60 cm of virtual space along the virtual corridor; see also axes in Figure 3E, F). The gain modulated trials were all performed in one session with trials interleaved such that 1:5 was gain modulated and 1:10 was gain modulated and uncued.

*Data analysis*

The virtual reality system was updated at a rate of 60 Hz. At each frame, custom code was executed that updated the state of the virtual environment and triggered events (e.g. dispense a reward) when appropriate. At the end of each frame, one line was added to the behavioral raw data file containing the necessary information for post-hoc data analysis (time since start of session, location, occurrence of a lick, etc.). Spatial displacement of the mouse in the virtual environment was calculated as the mean treadmill rotation recorded over the preceding 200 ms. Imaging and behavioral datafiles were aligned post-hoc. The behavioral datafile was matched to the imaging datafile by downsampling and interpolating such that the aligned dataset had the same number of frames. Binary behavioral events that could not be interpolated, such as licking or reward, were matched to the closest frame of the imaging dataset.

For classifying task-related neurons, we compared the activity within 25 cm blocks taken before and after the reward-zone onset for each trial (Rpre vs Rpost; $p<0.001$, Wilcoxon signed rank test; see Figure S1A). If $R_{pre}$ (-35 to -10 cm before the reward-zone onset) was significantly greater than $R_{post}$ (0 to 25 cm after the reward-zone onset), then the neuron decreases its activity at the reward zone and was categorized as corridor responsive. If $R_{post}$ was significantly greater than $R_{pre}$, then the neuron was considered reward-zone responsive (Figure S1).

We quantified the effect of locomotion on neuronal activity by using a locomotion modulation index (LMI), which is the difference between the ΔF/F0 during locomotion ($R_L$) and stationary (Rs) periods, normalized by the activity during both periods: LMI = ($R_L$ - Rs) ⁄ ($R_L$ + Rs) (see also Pakan et al., 2016).

To quantify the accuracy by which V1 activity could be classified based on the neuronal population activity, we used a template-matching decoder (Montijn et al., 2014), which compares the population activity per trial to response templates of the different experimental conditions (i.e. successful vs missed trials; visually-cued vs uncued trials). These templates are generated by taking the mean ΔF/F0 immediately prior to and within the reward zone (from 5 cm before the reward zone to the end of the reward zone) for each neuron in a single field of view, resulting in a template of population activity (Rt) per trial. The similarity of this template to the actual population activity (RP) for all other trials per condition is given by:

$$I_t = \frac{\sum_{i=1}^{N} R_i^P \cdot R_i^t}{|R^t| \cdot |R^P|},$$

where i indexes the N elements (neurons) of R. The similarity index I is calculated for all conditions and the decoded output is determined by taking the condition with the highest similarity to the template population activity. Decoder accuracy is given by the percentage of correctly decoded trials.

Multivariate analysis of covariance was performed for each stimulus condition (visually-cued and uncued trials) to test for the significance of a group effect of behavioral outcome (successful vs missed trials) with total trial time as a covariate for each neuron (see Figure S2).

*Statistics*

Error bars in all graphs indicate standard error of the mean (s.e.m.) and statistics were performed with two-tailed tests. Unless otherwise stated, for statistical tests comparing values across days or conditions (e.g. cued/uncued) for the same population of neurons, we used the Wilcoxon signed rank test (paired difference test). For statistical tests comparing measures across conditions that involved different underlying population of neurons, we used the Kruskal–Wallis test (one-way ANOVA on ranks). When analysing data from all neurons, we used the number of animals as our sample size because neuronal responses from the same mouse may be correlated and thus do not represent independent samples. Therefore, comparing measures across neurons, rather than across animals, may incorrectly inflate the degrees of freedom with the risk of false positive results for detecting significant differences.
