# Supplementary material for: The Impact of Visual Cues, Reward, and Motor Feedback on the Representation of Behaviorally Relevant Spatial Locations in Primary Visual Cortex
Source: Cell Rep. 2018 Sep 4;24(10):2521–8. doi: 10.1016/j.celrep.2018.08.010 (PMC6137817; doi:10.1016/j.celrep.2018.08.010)
Supplement: Document S2. Article plus Supplemental Information [file mmc2.pdf]

Cell Reports

Report

The Impact of Visual Cues, Reward, and Motor Feedback on the Representation of Behaviorally Relevant Spatial Locations in Primary Visual Cortex

Graphical Abstract

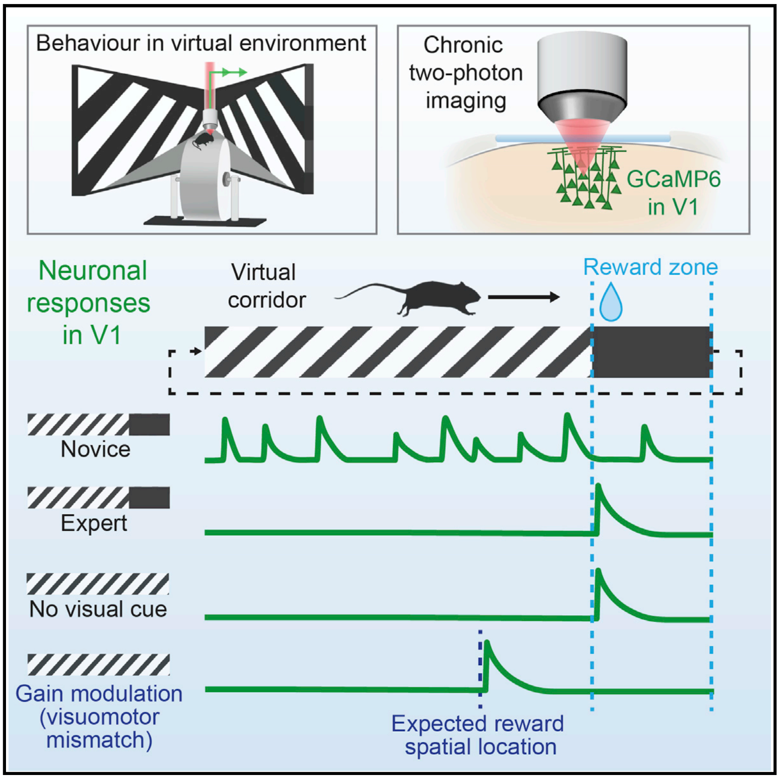

Highlights

- Most V1 L2/3 neurons show task-related activity after learning a rewarded task
- A subset of neurons became responsive to an expected reward location
- Without visual cues, behavioral and neuronal responses rely on self-motion signals
- With visual cues, behavioral and neuronal responses rely on visual information

Authors

Janelle M.P. Pakan, Stephen P. Currie, Lukas Fischer, Nathalie L. Rochefort

Correspondence

n.rochefort@ed.ac.uk

In Brief

Pakan et al. show that spatial locations that are relevant for a behavioral task are represented in the primary visual cortex. Both neuronal and behavioral responses to an expected reward location primarily rely on visual information. Without visual landmarks, both neuronal and behavioral responses are driven by self-motion derived information.

Pakan et al., 2018, Cell Reports *24*, 2521–2528
September 4, 2018 © 2018 The Author(s).
https://doi.org/10.1016/j.celrep.2018.08.010

CellPress

OPEN ACCESS
CellPress

The Impact of Visual Cues, Reward, and Motor Feedback on the Representation of Behaviorally Relevant Spatial Locations in Primary Visual Cortex

Janelle M.P. Pakan,[1,2,3,6] Stephen P. Currie,[1,6] Lukas Fischer,[1,4,6] and Nathalie L. Rochefort[1,5,7,*]
[1]Centre for Discovery Brain Sciences, Biomedical Sciences, Edinburgh EH8 9XD, UK
[2]Center for Behavioral Brain Sciences, Institute of Cognitive Neurology and Dementia Research, Otto-von-Guericke University, 39120 Magdeburg, Germany
[3]German Center for Neurodegenerative Diseases, 39120 Magdeburg, Germany
[4]McGovern Institute for Brain Research, Massachusetts Institute of Technology, Cambridge, MA 02139, USA
[5]Simons Initiative for the Developing Brain, University of Edinburgh, Edinburgh EH8 9XD, UK
[6]These authors contributed equally
[7]Lead Contact
*Correspondence: n.rochefort@ed.ac.uk
https://doi.org/10.1016/j.celrep.2018.08.010

SUMMARY

The integration of visual stimuli and motor feedback is critical for successful visually guided navigation. These signals have been shown to shape neuronal activity in the primary visual cortex (V1), in an experience-dependent manner. Here, we examined whether visual, reward, and self-motion-related inputs are integrated in order to encode behaviorally relevant locations in V1 neurons. Using a behavioral task in a virtual environment, we monitored layer 2/3 neuronal activity as mice learned to locate a reward along a linear corridor. With learning, a subset of neurons became responsive to the expected reward location. Without a visual cue to the reward location, both behavioral and neuronal responses relied on self-motion-derived estimations. However, when visual cues were available, both neuronal and behavioral responses were driven by visual information. Therefore, a population of V1 neurons encode behaviorally relevant spatial locations, based on either visual cues or on self-motion feedback when visual cues are absent.

INTRODUCTION

The ability to identify behaviorally relevant locations is critical for successful navigation through the environment and, ultimately, survival. This ability requires an estimation of location that can rely on positional cues, such as visual features of the environment, or on internal representations based on speed and direction of movement (Chen et al., 2013; Etienne and Jeffery, 2004; Tcheang et al., 2011; Tennant et al., 2018; Campbell et al., 2018). While it is well known that physical features of the visual world are represented by neuronal activity in the primary visual cortex (V1), recent studies have shown that self-motion-related information is also represented in V1 and can directly modulate visual responses (Erisken et al., 2014; Keller et al., 2012; Niell and Stryker, 2010; Pakan et al., 2016; Saleem et al., 2013). These results suggest that the visual cortex may combine motor-related and visual information to encode signals related to the spatial position of visual stimuli. Consistent with this hypothesis, it was shown that a subset of V1 neurons responds specifically to a given visual stimulus placed in one location along a virtual corridor and less to the same stimulus at another location (Fiser et al., 2016).

A representation of the spatial location of a visual cue in V1 (i.e., at an early stage of sensory information processing) may facilitate the perception of stimuli associated with danger or a reward at specific locations. However, it remains unknown whether V1 neurons represent spatial locations that are relevant for a behavioral task, such as the location associated with a reward, and whether spatial expectations would exclusively rely on visual cues or may also be triggered by self-motion signals alone. Previous studies have used visual discrimination tasks, in which mice learn to discriminate a rewarded visual stimulus from a non-rewarded one, to show that the representation of behaviorally relevant visual stimuli in V1 are enhanced with experience (Jurjut et al., 2017; Keller et al., 2017; Pakan et al., 2018; Poort et al., 2015). These results suggest that feedforward visual inputs are integrated with reward-related signals that have been shown to be present in V1 (Chubykin et al., 2013; Shuler and Bear, 2006). However, it is unclear whether visual, reward, and self-motion-related signals combine to activate V1 neurons in response to relevant spatial locations, such as a location associated with a reward.

In this study, we used two-photon calcium imaging in head-fixed mice placed in a virtual environment, to monitor the activity of V1 neurons before, during, and after mice learned to locate a reward on a virtual linear corridor. Mice had to lick at a given spatial location, demarcated by a visual cue, in order to receive a reward. We found that V1 neuronal activity correlated with behavioral responses: with training, most neurons became specifically responsive to the reward zone region of the virtual corridor. When the visual cue was removed but the reward remained at the same spatial location, we found that the expected reward location was represented by a subset of V1 neurons. We

This is an open access article under the CC BY license (http://creativecommons.org/licenses/by/4.0/).

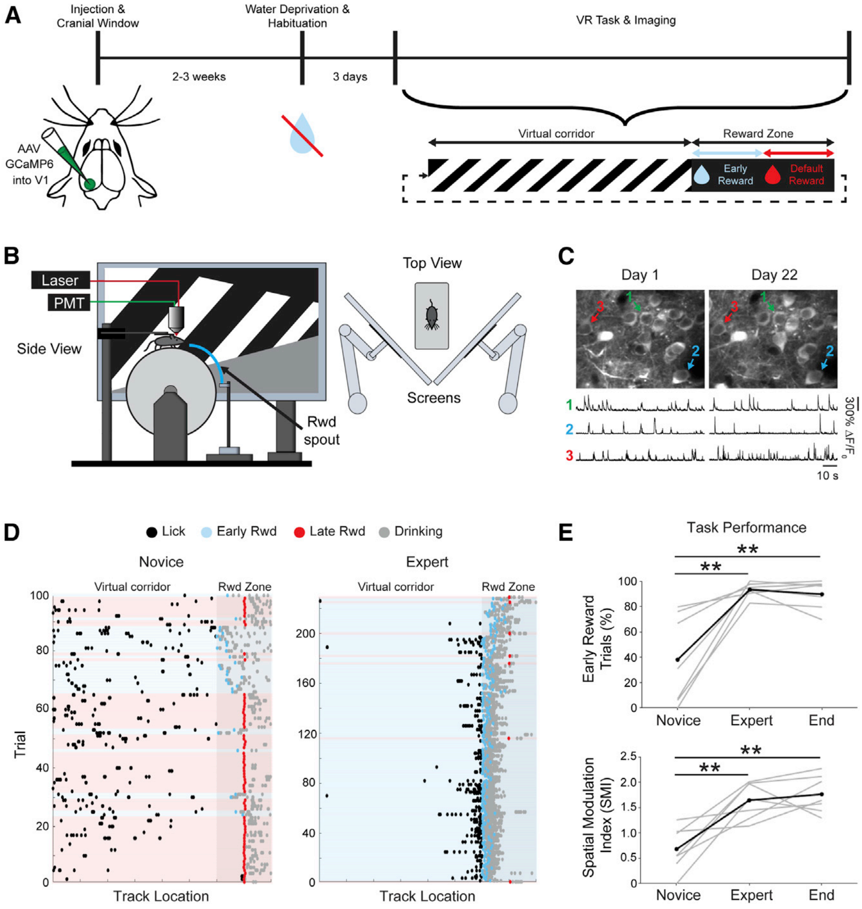

Figure 1. Mice Learn to Lick at a Specific Reward Location in a Visually Guided Task in a Virtual Environment

(A) Experimental timeline for chronic imaging of primary visual cortex (V1). The virtual corridor had a grating pattern on the walls and a reward zone demarcated by the transition to black walls. Early rewards were triggered by appropriate licking behavior within the first half of the reward zone, while a default reward was given on missed trials after the halfway point of the reward zone to facilitate learning.

(B) Schematic of the virtual reality system for two-photon imaging of V1 neurons during execution of the task. Mice were head-fixed above a cylindrical treadmill driving the virtual environment via a rotary encoder. The environment was displayed on two screens placed at 90° to one another and directly in front of the animal, covering the majority of the mouse visual field.

(C) Example images of the same GCaMP6f-labeled neurons in V1 during chronic imaging over days. The resulting changes in fluorescence ($\Delta F/F_0$) are shown for three example neurons.

(D) Raster plots of licking behavior during the task for a single mouse on novice (left) and expert (right) days. Each lick in the virtual corridor is represented by a black circle. Within the reward zone, three different lick types are illustrated: early reward (blue), default reward (red), and post-reward (drinking; gray).

(E) Task performance for novice, expert, and end-point imaging days is expressed either as the proportion of early rewards (top panel; novice to expert, $p = 0.002$; novice to end, $p = 0.006$, $n = 7$; Kruskal-Wallis test) or as a spatial modulation index (SMI) (success rate/proportion of trials that would be successful with a shuffled distribution of licking). A large SMI indicates that the animal was licking in a spatially discrete area surrounding the reward zone (SMI novice to expert, $p = 0.003$; SMI novice to end, $p = 0.002$, $n = 7$ mice; Kruskal-Wallis test; $^{**}p < 0.01$).

then manipulated the gain between treadmill rotation and the virtual environment to decouple visual information from self-motion feedback. Our results show that, in the absence of a visual cue, animal behavior and neural responses both rely on self-motion cues; however, in the presence of a visual cue indicating the reward location, visual input dominates self-motion cues.

RESULTS

We trained head-fixed mice to perform a visually guided task and used two-photon calcium imaging to assess changes in neuronal activity in V1 during learning (Figure 1). Seven mice were trained daily to perform a rewarded task in a virtual environment (Figures 1A and 1B) while we imaged the same population of layer 2/3 neurons, which expressed the genetically encoded calcium indicator GCaMP6f (Chen et al., 2013) (e.g., Figure 1C). The task required water-deprived mice to lick a spout for a water reward at a specific location along a virtual corridor (80 cm from the beginning of the corridor), which was indicated by a change in visual stimulus from an oriented grating pattern to black walls, referred to as the reward zone (Figure 1A). Once the animal entered the reward zone, within the first 20 cm (80–100 cm) it could lick for a water droplet (early reward, Figure 1A); this was considered a successful trial. To facilitate learning on missed trials, where a reward was not triggered by the mouse, animals were given a water droplet at a default location 20 cm beyond the reward zone onset (default reward, 100 cm, Figure 1A).

In the first training sessions, mice licked randomly along the length of the corridor but quickly learned to target their licking behavior to the reward zone region: they were considered "expert" at the task when they achieved a success rate of >75% early rewarded trials (e.g., Figure 1D). This criterion was achieved after an average of five sessions (range, 4–6 days) and was maintained through the remaining training days (Figure 1E). In this paradigm, it would be possible for the animals to adopt a strategy of licking constantly along the length of the corridor and still maintain a high success rate based on the percentage of early rewarded trials. To account for this, we calculated a spatial modulation index (SMI) (see Experimental Procedures) that significantly increased from 0.68 ± 0.16 on the novice day to 1.76 ± 0.14 by the end of the training sessions (Figure 1E, lower panel; $p = 0.002$, $n = 7$; Kruskal-Wallis test), indicating that mice learned to associate a water reward with

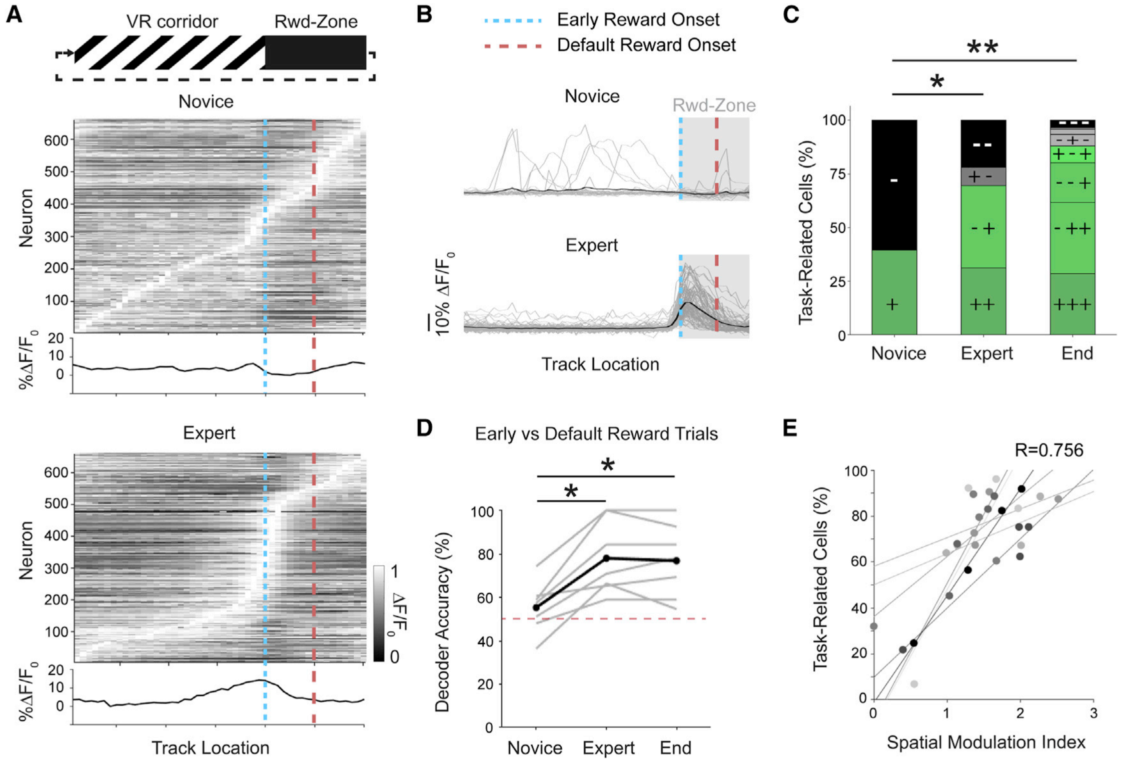

Figure 2. V1 Layer 2/3 Population Activity during Learning of the Visually Guided Task

(A) Normalized $\Delta F/F_0$ along the virtual corridor, plotted for all neurons on novice (top) and expert (bottom) days. Neurons are ordered by their maximal $\Delta F/F_0$ on the recording day. The mean of the normalized activity of all neurons is shown in the lower panels (n = 697 neurons from 7 mice). The early reward (blue; small dashed lines) and default reward (red; large dashed lines) onsets are demarcated.

(B) An example neuron that became task-responsive (significant change in response before and after the reward zone) between novice (top) and expert (bottom) days. $\Delta F/F_0$ for individual trials (gray traces) and average across all trials (black trace) are shown (see also Figure S2B).

(C) Bar chart showing the evolution of the V1 population response during learning. At each stage (novice, expert, and end-point days), neurons were classified as either task-responsive (+) or not (–) (i.e., neurons that remain task-responsive at each stage of learning were denoted +++; novice to expert, p = 0.025; novice to end, p = 0.010, n = 7; Kruskal-Wallis test; *p < 0.05, **p < 0.01).

(D) Accuracy of a template matching decoder using V1 population activity to predict behavioral outcome of either successful (early rewarded) or missed (default rewarded) trials. The decoder accuracy increased with training (novice to expert, p = 0.015; novice to end, p = 0.015, n = 7; Wilcoxon signed rank; *p < 0.05). Dashed line indicates chance level.

(E) Correlation between the proportion of task-related neurons and the success rate (SMI) of the task (Pearson's correlation coefficient). Each data point represents one animal on one day of training. Data points from the same animal have the same shade of gray and are fit by least-squares regression lines.

See also Figures S1 and S2.

the visually cued location and consequently produce spatially confined licking behavior.

Most V1 Layer 2/3 Neurons Display Task-Related Responses after Learning

On the first day of training (novice), the maximal response of neurons ranged across all locations along the corridor; however, by the expert day (success rate, >75%), a large proportion of peak responses were centered around the reward zone transition (Figures 2A, 2B, and S2B). We identified task-related neurons as those having a significant change in response before (R_{pre}) compared to after (R_{post}) the reward zone onset (R_{pre} versus R_{post}: $p < 0.001$, Wilcoxon signed rank test; Figure S1A). We found that, with training, most neurons became specifically responsive to the reward zone transition (percentage of task-related cells, 40% ± 12% novice, 88% ± 3% end of training; p = 0.010, n = 7; Kruskal-Wallis test; Figures 2A–2C; see also Figure S2B). Consequently, when we utilized a template matching decoder (Montijn et al., 2014; see Supplemental Experimental Procedures), using neuronal population activity to predict behavioral outcome by differentiating between successful trials (early rewarded) and missed trials (default rewarded), the decoder accuracy significantly increased from novice to expert days (decoder accuracy, 55% ± 5% novice, 77% ± 6% end of training; p = 0.015, n = 7; Wilcoxon signed rank; Figure 2D). Accordingly, the proportion of task-related neurons correlated with the behavioral success rate (quantified by the SMI; Figure 2E).

As the mice learned the task, they became faster at performing it and more consistent in their execution (Figure S2A). We thus tested whether the task-related responses observed on expert days were due to an entrainment effect of a stereotypic trial time. We found consistent responses at the reward zone onset

even for the slowest and fastest trial times, which could differ from each other by more than an order of magnitude (Figure S2B). The task-related responses were thus more consistent across distance than time and did not reflect stimulus entrainment (Figures S2D–S2F).

The large proportion of V1 task-related neurons on expert day included a variety of responses with neurons either decreasing or increasing their activity at the reward zone (Figures S1A and S1B). Neurons decreasing their activity included neurons that were responsive to the oriented grating along the corridor and decreased their activity at the reward zone onset (transition to black walls; corridor responsive; 39%), as well as neurons that decreased their activity with lower running speed (locomotion responsive; 12%; Figures S1B and S1C). Neurons increasing their activity at the reward zone onset included a small proportion of neurons responding to licking independently of the reward (lick responsive; 5%) and reward zone-related neurons (21%; Figures S1B and S1C). We then tested the relative contribution of the visual cue (black walls) and self-motion-related cues to the reward zone-related responses.

Neuronal and Behavioral Responses at a Reward Location in the Absence of a Visual Cue

After reaching the expert day, all seven mice were tested on an additional corridor configuration (phase 2) in which the reward zone remained at the same distance along the virtual corridor but was no longer "cued" by a visual landmark (i.e., the black corridor walls demarcating the reward zone were removed; see Figure 3A). In these uncued trials, animals still had to lick at the same physical location along the corridor to receive the reward and be considered a successful trial. However, as before, if they did not lick successfully they also received a later reward at the default location (see Figure 1A). On the first day without a visual cue, the success rate was 44% ± 4% on uncued trials, and after an average of six sessions (range, four to seven), mice reached the 75% ± 4% success rate criteria to be considered expert (Figure 3B).

From the population responses in V1 layer 2/3, we identified neurons responding at the reward location in both visually cued as well as uncued trials ($R_{post} > R_{pre}$: $p < 0.001$, Wilcoxon signed rank test, in both conditions). An example neuron is shown in Figure 3A (see also Figure S2C). We thus excluded neurons that were specifically responding to the grating offset (off response). On the novice day without a visual cue, 7% of neurons specifically responded to the reward zone in both cued and uncued trials. However, by the expert day this proportion had doubled (15%). On the first uncued day, neurons showed distinct responses to successful cued and uncued trials, whereas by the expert day, responses to the visually cued and uncued trials were similar (Figure 3C, upper panel). When we utilized a template matching decoder to predict whether a given successful trial was either visually cued or uncued from all neuronal responses, the accuracy of the decoder significantly decreased from the novice to expert day (Figure 3D; decoder accuracy: novice, 90% ± 4%; expert, 73% ± 5%, p = 0.015, n = 7; Wilcoxon signed rank). This result was consistent with the increased proportion of neurons showing corresponding responses to cued and uncued trials, making these conditions less distinguishable.

Reward zone responses in the absence of the visual landmark may result from multiple variables: the licking behavior, the time from trial onset (through an entrainment effect), reward consumption, the spatial location of the reward, or a combination of these signals. We tested the response of this neuronal population to licking behavior by analyzing licks that occurred along the virtual corridor (outside the reward zone): the activity of the neurons during licking was not significantly different from non-licking periods (mean $\Delta F/F0$: licking, 0.32 ± 0.08; non-licking, 0.31 ± 0.07; p = 0.535; Wilcoxon signed rank). We then assessed the contribution of time (Figure S2C) and found that neuronal responses in uncued trials were more consistent across distance than time and did not reflect stimulus entrainment (Figures S2D and S2F). Next, we tested the response to reward consumption. The population of neurons that developed the reward zone-specific responses by the expert day showed a peak response at the reward event for both successful (early reward) and missed (default reward) trials, in which reward occurred at different spatial locations (Figure 3C), indicating that this neuronal population was responsive to the reward. This suggests that individual neuronal responses could reflect either the reward event itself or the reward associated to a specific spatial location.

Neuronal and Behavioral Responses to a Reward Location Based on Self-Motion-Related Information

To further investigate whether responses in V1 could specifically represent an expected location of a reward, we altered the gain relating the rotation of the cylindrical treadmill to the progression of the virtual corridor. In this last phase of the experiment, we used three expert trained mice and reduced the gain from 1 to 0.75 in a subset of trials. In this condition, the expected (i.e., trained) reward location was at 80 cm of distance traveled by the mice on the treadmill; however, this physical distance now correlated to only 60 cm in virtual space, along the virtual corridor (Figure 3E). If the mice were relying on motor-derived self-motion cues alone, they would lick at 80 cm of physical distance traveled on the treadmill (corresponding to 60 cm on the virtual corridor). If the mice were relying on the virtual corridor cues (such as the number of stripes), they would lick at 80 cm in virtual space (corresponding to 107-cm physical distance traveled). In these trials, the reward was given at 80 cm in virtual space along the virtual corridor, therefore after the expected reward location based on physical distance along the treadmill (see also Supplemental Experimental Procedures). We found a subset of neurons that showed significant gain-modulated responses on the uncued trials (Figure 3E; gain-modulated cells, 10% of the population). On average, these neurons had a peak response approximately midway between the expected reward onset and the actual reward onset (Figure 3F). We assessed the contribution of time to these neuronal responses and found that neuronal responses in gain-modulated trials were less variable across distance than time (Figures S2D and S2E). In most of these uncued gain-modulated trials, the mice also licked at the expected reward location (Figure 3G). When the mice did not lick at the expected reward location, the response amplitude of these neurons (between the expected reward onset and the actual reward onset) was decreased by two-thirds, without

OPEN ACCESS
CellPress

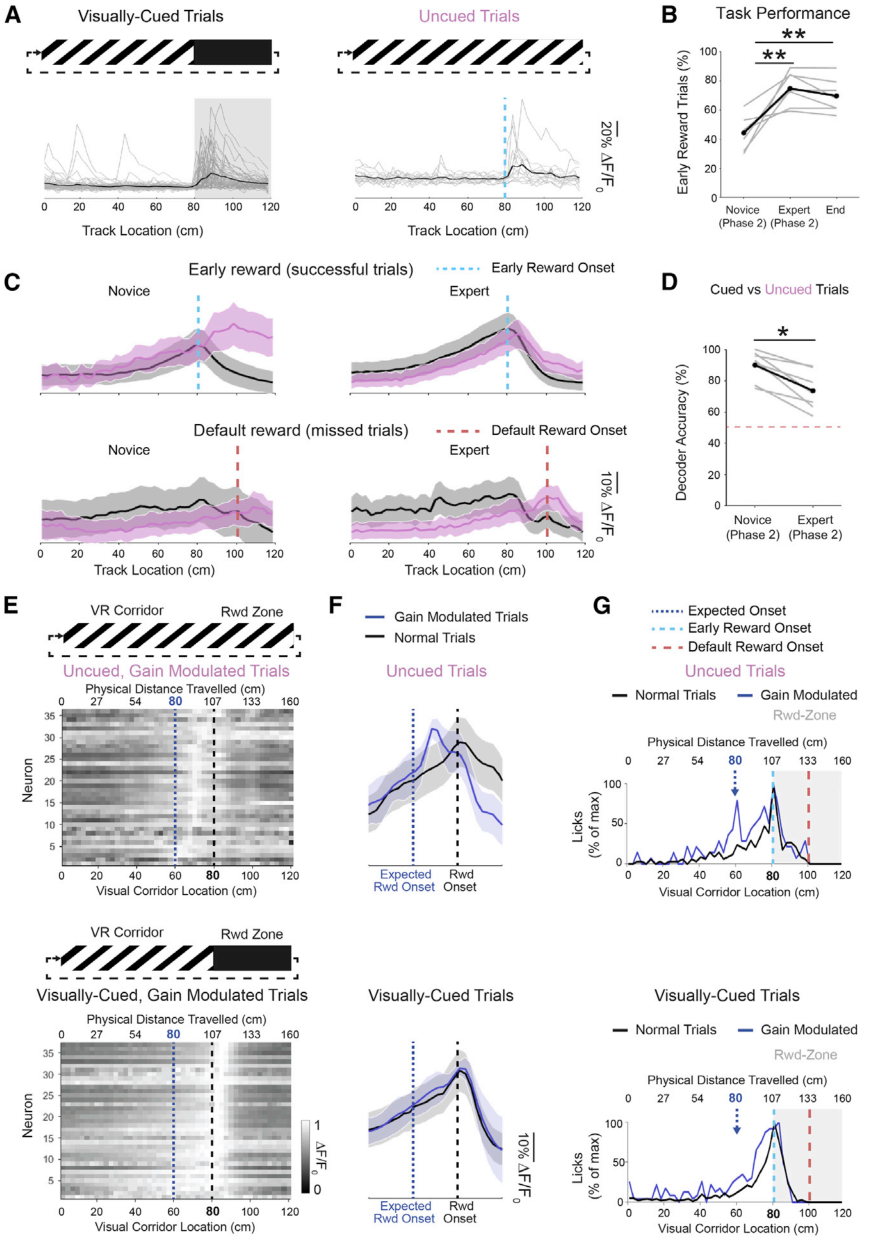

Figure 3. After Learning, V1 Neurons Respond to a Rewarded Spatial Location, Even in the Absence of Visual Cues

(A) After mice reached expert criteria on the visually cued task (left), they were subsequently (phase 2) exposed to a subset of trials (one in five) where the visual landmark (black walls) for the reward zone were removed (right; uncued trials; dashed line indicates reward zone onset). $\Delta F/F_0$ is shown for an example neuron after reaching expert criteria on the uncued task (expert phase 2) for individual trials (gray traces) and average across all trials (black trace), highlighting reward zone-specific activity, even in the absence of a visual cue.

(B) Task success rate (% of early rewards) for uncued trials for the novice, expert, and end-point days of phase 2 (novice to expert, $p = 0.004$; novice to end, $p = 0.006$, $n = 7$; Kruskal-Wallis test; $^{**}p < 0.01$).

(C) Mean $\Delta F/F_0$ traces and SEM of all neurons classified as responding to the reward location on both the cued and the uncued trials on the expert day ($R_{post} > R_{pre}$, $p < 0.001$, Wilcoxon signed rank test). Activity of these neurons is shown for both novice (left) and expert (right) days of phase 2 ($n = 104/697$ neurons from 7 mice). Within each day, trials are sub-divided into successful (early rewarded; upper panels) and missed (default rewarded; lower panels) trials. The early reward (blue; small dashed lines) and default reward (red; large dashed lines) onsets are demarcated.

(D) Accuracy of a template matching decoder using V1 population activity to predict trial type (cued versus uncued) on either novice or expert days of phase 2 ($p = 0.015$, $n = 7$; Wilcoxon signed rank; $^{*}p < 0.05$). Dashed line indicates chance level.

(E) In phase 3, the gain of the virtual reality system was manipulated from 1 to 0.75 in a subset of trials (one in five). Both the physical distance traveled on the cylindrical treadmill (upper x axis) and the distance in virtual space along the virtual corridor (lower x axis) are indicated. The expected reward onset based on physical distance traveled on the treadmill is indicated by the blue dashed line, while the actual reward onset based on the distance along the virtual corridor is indicated by the black dashed line. Normalized $\Delta F/F_0$ is shown for all gain-modulated neurons, ordered by their maximal $\Delta F/F_0$ response on the recording day ($n = 38/362$ neurons from 3 mice).

(F) Mean $\Delta F/F_0$ traces and SEM of all neurons classified as gain modulated. Responses on uncued (upper) and visually cued (lower) trials are shown for both normal (gain = 1; black lines) and gain-modulated (gain = 0.75; blue lines) trials. The expected spatial location of the reward (blue dashed line) and actual reward onset (black dashed line) are marked for reference.

(G) The normalized pre-reward licking behavior along the virtual corridor (all licks recorded before receiving the reward were summed across all mice then normalized to % of max licking) are shown for both uncued (upper) and visually cued (lower) trials. The trials are further separated into normal (gain = 1; black lines) and gain modulated (gain = 0.75; blue lines). The expected (dark blue; small dashed arrow), early (light blue; medium dashed line), and default (red; large dashed line) reward onsets are marked for reference. Same x axes as in (E).

See also Figure S2.

any clear peak. These results indicate that the gain-modulated neuronal responses correlate with the behavioral expectation of a reward at this specific location (see also Figure S2F). Therefore, in the absence of a visual cue (black walls), mice determined reward location based on self-motion-related information.

When Available, Visual Information Drives Neuronal and Behavioral Responses to Reward Location

In the gain-modulated visually cued trials, the visual cue was visible ahead of the mouse when it reached the expected reward location based on physical distance traveled. Interestingly, in these trials, the gain-modulated neurons showed no significant

response near the expected reward onset. Instead, these neurons responded at the actual reward location, which was demarcated by the visual cue (Figures 3E and 3F), indicating that in these trials visual inputs dominated the responses of these neurons. Correspondingly, mice also licked at the actual reward location indicated by the landmark (Figure 3G). These results indicate that, in the presence of the visual cue, mice primarily relied on visual information to identify the reward location. Similarly, visual inputs related to the landmark dominated the responses of V1 neurons.

DISCUSSION

Our results demonstrate a recruitment of the majority of V1 layer 2/3 neurons to task-relevant activity while animals learned to locate a reward in a virtual environment. We show that a subset of neurons responded to the specific spatial location associated with an expected reward. In the absence of a visual cue, this neuronal representation of reward location relied on self-motion-related inputs and correlated with behavioral outcome. However, when visual cues were available, both neuronal and behavioral responses were driven by visual information. Importantly, these responses were specific to a rewarded spatial location (i.e., a behaviorally relevant location) and appeared after learning: thus, they correspond to an expectation of a reward at a given location. This differs from a cognitive map, or a comprehensive spatial mapping of the environment, as described in CA1 place cells: in our experimental conditions, we did not observe place cell-like mapping of spatial locations all along the virtual corridor.

In the absence of visual landmarks, mice can use different strategies to determine the reward location. One such strategy would be to estimate the distance traveled based on optic flow information provided by the pattern of the virtual corridor. However, when we changed the gain between physical and virtual space, mice licked at the expected location based on the physical distance they had run on the treadmill, as opposed to using optic flow information. The evaluation of the distance to the reward location was thus based on locomotor-related feedback information. Our results are consistent with the hypothesis that, in the absence of visual cues, mice are able to estimate the distance toward a reward based on self-motion feedback information. This result is in line with previous studies showing that mice can use path integration mechanisms to estimate location (Van Cauter et al., 2013; Etienne and Jeffery, 2004; Tennant et al., 2018; Campbell et al., 2018).

While the encoding of spatial information has been extensively characterized in the hippocampal formation (Dombeck et al., 2010; Hartley et al., 2013), our results show that a subset of V1 neurons receive inputs related to spatial location. This signal could originate from a number of sources. It could be conveyed by top-down cortico-cortical inputs. For example, neurons in the retrosplenial cortex have been shown to encode spatial and navigational signals (Mao et al., 2017). Since retrosplenial cortex is one of the major sources of input to V1 (Leinweber et al., 2017), it is possible that spatial representations present in retrosplenial cortex are transmitted to a subset of V1 neurons. Another potential source of self-motion-related inputs is the anterior cingulate cortex and premotor areas (A24b/M2). These areas were shown to convey motor-related excitatory inputs to V1 neurons and are thought to carry a prediction of visual flow based on self-motion information (Leinweber et al., 2017). Spatial signals could also be conveyed to V1 through subcortical inputs. For example, the lateral posterior nucleus of the thalamus has been shown to convey locomotion-related and contextual signals to V1 neurons (Roth et al., 2016). The encoding of behaviorally important spatial locations could either occur in the aforementioned cortical and subcortical areas and be transmitted to V1, or encoding could occur in V1 itself since previous studies have shown neuronal responses to running speed (Erisken et al., 2014; Keller et al., 2012; Pakan et al., 2016; Saleem et al., 2013) as well as to reward-timing in mouse V1 (Chubykin et al., 2013). Together, these recent studies, and our current results, indicate that information about reward anticipation and motor feedback cues are available directly to V1 and may be used by this primary sensory area to facilitate visual identification of behaviorally relevant environmental cues, which has direct implications for navigation and more generally for visual perception.

Our results further show that visual input overrides self-motion-derived estimates of location in V1 neurons. Potential underlying mechanisms may include visual excitatory inputs that dominate self-motion ones or visual inputs that inhibit spatially related information. This process may occur either within V1 or in other brain areas. For instance, it has been shown that the majority of place cells in the hippocampus require visual input to display spatially localized firing within a visual virtual environment (Chen et al., 2013). It was suggested that visual inputs may be conveyed to place cells through neurons found in the subiculum and entorhinal cortex (Hartley et al., 2000; Lever et al., 2009). Spatial representations arising in the hippocampal formation may then be transmitted to V1 through cortical or subcortical pathways. Additionally, V1-projecting anterior cingulate cortex axons convey spatially modulated signals and show visual stimuli predictive activity, suggesting that anterior cingulate cortex serves as a source of predictions of visual input to V1 (Fiser et al., 2016). Further studies are needed to determine the circuit mechanisms underlying the relative contribution of visual and self-motion-related inputs to the representation of relevant spatial locations in V1.

Altogether, our results show that neuronal activity in adult V1 is highly dynamic and is shaped by the behavioral significance of task-related information, including relevant spatial locations. Both neuronal and behavioral responses primarily rely on visual information, when a visual cue is available. However, in the absence of visual cues, the animal behavior as well as neuronal responses can be driven by self-motion-derived information.

EXPERIMENTAL PROCEDURES

Animals

All animal experiments were approved by the Animal Welfare and Ethical Review Board (AWERB) of the University of Edinburgh and were performed under a project license granted by the UK Home Office, and conformed with the UK Animals (Scientific Procedures) Act 1986 and the European Directive 86/609/EEC on the protection of animals used for experimental purposes.

Seven male and female mice with a C57BL/6 background (*Sst*$^{tm2.1(cre)Zjh}$/J [RRID:IMSR_JAX:013044] cross-bred with B6.Cg-*Gt(ROSA)26Sor*$^{tm14(CAG-tdTomato)Hze}$/J [RRID:IMSR_JAX:007914]; Jackson

Laboratory, ME, USA), aged 6–7 weeks, were used for the experiments. Animals were group housed in a reverse day/night cycle.

Surgical Procedures

For cranial window implantation and virus injection, mice were anaesthetized with isoflurane (4% for induction and 1%–2% maintenance during surgery) and mounted on a stereotaxic frame (David Kopf Instruments, CA, USA). Eye cream was applied to protect the eyes (Bepanthen; Bayer, Germany), and analgesics and anti-inflammatory drugs were injected subcutaneously (Vetergesic, buprenorphine, 0.1 mg/kg of body weight; carprofen, 0.15 mg; and dexamethasone, 2 μg). A section of scalp was removed and the underlying bone cleaned before a craniotomy (around 2 × 2 mm) was made over the left V1 (centered 2.5 mm lateral and 0.5 mm anterior to lambda). Then adeno-associated virus (AAV) (AAV1.Syn.GCaMP6f.WPRE.SV40; University of Pennsylvania Vector Core, PA, USA) was injected, to drive the expression of the fluorescent calcium indicator GCaMP6f in all neurons, using a pipette with 20-μm tip diameter (Nanoject; Drummond Scientific, PA, USA) at a speed of 10 nL min^{-1} at three different depths (around 250, 400, and 600 μm deep; 50 nL per site). The craniotomy was then sealed with a glass coverslip and fixed with cyanoacrylate glue. A custom-built head post was implanted on the exposed skull with glue and cemented with dental acrylic (Paladur; Heraeus Kulzer, Germany). Animals were returned to their home cage for 2–3 weeks to allow for virus expression and clearing of the imaging window (Holtmaat et al., 2009) before habituation and imaging.

Virtual Reality System

Animals were trained on a virtual reality system consisting of two angled computer screens (Figure 1B), a cylindrical treadmill, head fixation system, and a reward spout. The computer screens (51 × 29 cm; Dell, UK) were placed at a 90° angle in front of the animal covering the majority of its field of view. A cylindrical polystyrene treadmill (20 cm diameter, 7.5 cm wide) was mounted on a central axle with an incremental rotary encoder (E6-2500-472-IE; Pewatron, Switzerland). A microcontroller (Arduino Uno) received rotational displacement information from the encoder and forwarded it the virtual reality software. The reward spout (59-8636; Harvard Apparatus, UK) was fitted with a capacitive touch sensor (SEN-12041; Sparkfun, CO, USA) to detect animal licking behavior and put into place at the beginning of each session, such that the animal was always able to reach it. Reward release was controlled by a pinch-valve (225PNC1-21; NResearch, NJ, USA) that dispensed 4- to 8-μL boluses per instance. The MATLAB-based package ViRMEn (Aronov and Tank, 2014) combined with custom-written code was used to design and run the presentation of the virtual environment and collect related data (see Supplemental Experimental Procedures).

Visually Guided Rewarded Task

Mice were water deprived (see Supplemental Experimental Procedures) and water rewards could either be self-initiated by licking in the first half of the reward zone (early, successful trial), or were dispensed at a default location at the halfway point of the reward zone (late, missed trial). Behavioral training was divided into three phases. For each phase, the first day was taken as the "novice" day. The animals were considered "expert" and promoted to the next phase of training, when successful trials made up >75% of the total trials. For phase 1, the mice were exposed to a single virtual corridor condition with the reward zone visually cued by black corridor walls. Phase 2 introduced uncued trials on every fifth trial, where the rules for reward remained the same but the black corridor walls were removed. For three mice, an additional phase 3 was performed where in a single session the gain relating the rotation of the cylindrical treadmill to the progression in the virtual corridor was reduced from 1 to 0.75 (see Supplemental Experimental Procedures).

Two-Photon Calcium Imaging

Two-photon calcium imaging was performed in head-fixed mice that ran freely on the cylindrical treadmill (Figure 1B) (Dombeck et al., 2007) using a custom-built resonant scanning two-photon microscope with a Ti:sapphire pulsing laser (Chameleon Vision-S; Coherent, CA, USA; <70-fs pulse width, 80-MHz repetition rate) tuned to 920 nm. Images were acquired at 40 Hz (using 40×, 0.8 numerical aperture [NA], or 25×, 1.05 NA; Nikon) with custom-programmed LabVIEW-based software (version 8.2; National Instruments, UK). Imaging was done at a single L2/3 focal plane per mouse across multiple days, at cortical depths between 150 and 275 μm. Laser power at the brain surface was kept below 50 mW. Chronic imaging of the same field of view across days was carried out for the duration of the visually guided reward task.

Data Analysis

Images were analyzed as previously described (Pakan et al., 2016). Briefly, we used discrete Fourier two-dimensional (2D)-based image alignment for motion correction of image frames (SIMA 1.3.2) (Kaifosh et al., 2014). Regions of interest (ROIs) corresponding to neuronal cell bodies were selected manually and aligned across days. Pixel intensity within each ROI was averaged to create a raw fluorescence time series F(t). Baseline fluorescence F_0 was computed for each neuron by taking the fifth percentile of the smoothed F(t) (1-Hz low-pass, zero-phase, 60th-order FIR filter) and the change in fluorescence relative to baseline ($\Delta F/F_0$) was calculated. In order to remove neuropil contamination, we used nonnegative matrix factorization (NMF), as implemented in FISSA (Keemink et al., 2018). All further analyses were performed using custom-written scripts in MATLAB (MathWorks, MA, USA), which are freely available via the Rochefort Lab GitHub repository (https://github.com/rochefort-lab/Pakanetal_CellReport2018).

To calculate the SMI of licking, the licks of each trial were randomly permuted, and the proportion of trials in which at least one lick was inside the reward zone was determined. This was repeated 1,000 times, and the mean success rate of the shuffled distribution was calculated. The SMI value was calculated by dividing the original success rate (early rewarded trials/total number of trials) by the mean of the shuffled distribution. If the animal licks few times but in the right spot, this number will be high (>1). In contrast, if the animal licks in a spatially indiscriminate pattern, the number will approach 1. If the animal licks often but keeps missing the reward zone, the SMI will be <1.

Gain-modulated neurons were defined by meeting two criteria: a maximal response that fell within a 25-cm bin surrounding the reward zone onset (−5 to 20 cm from onset) when responses were averaged across all normal trials (gain = 1) as well as a maximal response that fell within a 25-cm bin surrounding the expected reward zone onset (−5 to 20 cm from expected onset) when responses were averaged across all gain-modulated trials (gain = 0.75).

SUPPLEMENTAL INFORMATION

Supplemental Information includes Supplemental Experimental Procedures and two figures and can be found with this article online at https://doi.org/10.1016/j.celrep.2018.08.010.

ACKNOWLEDGMENTS

We thank Ian Duguid, Matt Nolan, and their research groups for advice on behavioral training, virtual reality, and recordings in awake mice. We thank Nathalie Dupuy for preparing the available github repository. We thank the GENIE Program and the Janelia Research Campus, specifically V. Jayaraman, R. Kerr, D. Kim, L. Looger, and K. Svoboda, for making GCaMP6 available. This work was funded by the Wellcome Trust and the Royal Society (Sir Henry Dale Fellowship to N.L.R.), the Marie Curie Actions of the European Union's FP7 Program (MC-CIG 631770 to N.L.R. and IEF 624461 to J.M.P.P.), the Shirley Foundation, the Patrick Wild Center, and the RS MacDonald Charitable Trust Seedcorn Grant (to N.L.R.), the Simons Initiative for the Developing Brain (to N.L.R.), and the European Regional Development Fund (ERDF: Center for Behavioral Brain Sciences to J.M.P.P.).

AUTHOR CONTRIBUTIONS

L.F., S.P.C., and N.L.R. designed the experiments. L.F. and S.P.C. performed the experiments. J.M.P.P. analyzed the data with input from all authors. J.M.P.P. and N.L.R. wrote the manuscript with input from all authors.

DECLARATION OF INTERESTS

The authors declare no competing interests.

Received: April 19, 2018
Revised: June 8, 2018
Accepted: August 6, 2018
Published: September 4, 2018

REFERENCES

Aronov, D., and Tank, D.W. (2014). Engagement of neural circuits underlying 2D spatial navigation in a rodent virtual reality system. Neuron *84*, 442–456.

Campbell, M.G., Ocko, S.A., Mallory, C.S., Low, I.I.C., Ganguli, S., and Giocomo, L.M. (2018). Principles governing the integration of landmark and self-motion cues in entorhinal cortical codes for navigation. Nat. Neurosci. *21*, 1096–1106.

Chen, G., King, J.A., Burgess, N., and O'Keefe, J. (2013). How vision and movement combine in the hippocampal place code. Proc. Natl. Acad. Sci. USA *110*, 378–383.

Chubykin, A.A., Roach, E.B., Bear, M.F., and Shuler, M.G.H. (2013). A cholinergic mechanism for reward timing within primary visual cortex. Neuron *77*, 723–735.

Dombeck, D.A., Khabbaz, A.N., Collman, F., Adelman, T.L., and Tank, D.W. (2007). Imaging large-scale neural activity with cellular resolution in awake, mobile mice. Neuron *56*, 43–57.

Dombeck, D.A., Harvey, C.D., Tian, L., Looger, L.L., and Tank, D.W. (2010). Functional imaging of hippocampal place cells at cellular resolution during virtual navigation. Nat. Neurosci. *13*, 1433–1440.

Erisken, S., Vaiceliunaite, A., Jurjut, O., Fiorini, M., Katzner, S., and Busse, L. (2014). Effects of locomotion extend throughout the mouse early visual system. Curr. Biol. *24*, 2899–2907.

Etienne, A.S., and Jeffery, K.J. (2004). Path integration in mammals. Hippocampus *14*, 180–192.

Fiser, A., Mahringer, D., Oyibo, H.K., Petersen, A.V., Leinweber, M., and Keller, G.B. (2016). Experience-dependent spatial expectations in mouse visual cortex. Nat. Neurosci. *19*, 1658–1664.

Hartley, T., Burgess, N., Lever, C., Cacucci, F., and O'Keefe, J. (2000). Modeling place fields in terms of the cortical inputs to the hippocampus. Hippocampus *10*, 369–379.

Hartley, T., Lever, C., Burgess, N., and O'Keefe, J. (2013). Space in the brain: how the hippocampal formation supports spatial cognition. Philos. Trans. R. Soc. Lond. B Biol. Sci. *369*, 20120510.

Holtmaat, A., Bonhoeffer, T., Chow, D.K., Chuckowree, J., De Paola, V., Hofer, S.B., Hübener, M., Keck, T., Knott, G., Lee, W.-C.A., et al. (2009). Long-term, high-resolution imaging in the mouse neocortex through a chronic cranial window. Nat. Protoc. *4*, 1128–1144.

Jurjut, O., Georgieva, P., Busse, L., and Katzner, S. (2017). Learning enhances sensory processing in mouse V1 before improving behavior. J. Neurosci. *37*, 6460–6474.

Kaifosh, P., Zaremba, J.D., Danielson, N.B., and Losonczy, A. (2014). SIMA: Python software for analysis of dynamic fluorescence imaging data. Front. Neuroinform. *8*, 80.

Keemink, S.W., Lowe, S.C., Pakan, J.M.P., Dylda, E., van Rossum, M.C.W., and Rochefort, N.L. (2018). FISSA: a neuropil decontamination toolbox for calcium imaging signals. Sci. Rep. *8*, 3493.

Keller, G.B., Bonhoeffer, T., and Hübener, M. (2012). Sensorimotor mismatch signals in primary visual cortex of the behaving mouse. Neuron *74*, 809–815.

Keller, A.J., Houlton, R., Kampa, B.M., Lesica, N.A., Mrsic-Flogel, T.D., Keller, G.B., and Helmchen, F. (2017). Stimulus relevance modulates contrast adaptation in visual cortex. eLife *6*, e21589.

Leinweber, M., Ward, D.R., Sobczak, J.M., Attinger, A., and Keller, G.B. (2017). A sensorimotor circuit in mouse cortex for visual flow predictions. Neuron *95*, 1420–1432.e5.

Lever, C., Burton, S., Jeewajee, A., O'Keefe, J., and Burgess, N. (2009). Boundary vector cells in the subiculum of the hippocampal formation. J. Neurosci. *29*, 9771–9777.

Mao, D., Kandler, S., McNaughton, B.L., and Bonin, V. (2017). Sparse orthogonal population representation of spatial context in the retrosplenial cortex. Nat. Commun. *8*, 243.

Montijn, J.S., Vinck, M., and Pennartz, C.M.A. (2014). Population coding in mouse visual cortex: response reliability and dissociability of stimulus tuning and noise correlation. Front. Comput. Neurosci. *8*, 58.

Niell, C.M., and Stryker, M.P. (2010). Modulation of visual responses by behavioral state in mouse visual cortex. Neuron *65*, 472–479.

Pakan, J.M., Lowe, S.C., Dylda, E., Keemink, S.W., Currie, S.P., Coutts, C.A., and Rochefort, N.L. (2016). Behavioral-state modulation of inhibition is context-dependent and cell type specific in mouse visual cortex. eLife *5*, e14985.

Pakan, J.M., Francioni, V., and Rochefort, N.L. (2018). Action and learning shape the activity of neuronal circuits in the visual cortex. Curr. Opin. Neurobiol. *52*, 88–97.

Poort, J., Khan, A.G., Pachitariu, M., Nemri, A., Orsolic, I., Krupic, J., Bauza, M., Sahani, M., Keller, G.B., Mrsic-Flogel, T.D., and Hofer, S.B. (2015). Learning enhances sensory and multiple non-sensory representations in primary visual cortex. Neuron *86*, 1478–1490.

Roth, M.M., Dahmen, J.C., Muir, D.R., Imhof, F., Martini, F.J., and Hofer, S.B. (2016). Thalamic nuclei convey diverse contextual information to layer 1 of visual cortex. Nat. Neurosci. *19*, 299–307.

Saleem, A.B., Ayaz, A., Jeffery, K.J., Harris, K.D., and Carandini, M. (2013). Integration of visual motion and locomotion in mouse visual cortex. Nat. Neurosci. *16*, 1864–1869.

Shuler, M.G., and Bear, M.F. (2006). Reward timing in the primary visual cortex. Science *311*, 1606–1609.

Tcheang, L., Bülthoff, H.H., and Burgess, N. (2011). Visual influence on path integration in darkness indicates a multimodal representation of large-scale space. Proc. Natl. Acad. Sci. USA *108*, 1152–1157.

Tennant, S.A., Fischer, L., Garden, D.L.F., Gerlei, K.Z., Martinez-Gonzalez, C., McClure, C., Wood, E.R., and Nolan, M.F. (2018). Stellate cells in the medial entorhinal cortex are required for spatial learning. Cell Rep. *22*, 1313–1324.

Van Cauter, T., Camon, J., Alvernhe, A., Elduayen, C., Sargolini, F., and Save, E. (2013). Distinct roles of medial and lateral entorhinal cortex in spatial cognition. Cereb. Cortex *23*, 451–459.

Cell Reports, Volume *24*

Supplemental Information

The Impact of Visual Cues, Reward, and Motor Feedback on the Representation of Behaviorally Relevant Spatial Locations in Primary Visual Cortex

Janelle M.P. Pakan, Stephen P. Currie, Lukas Fischer, and Nathalie L. Rochefort

SUPPLEMENTAL FIGURES

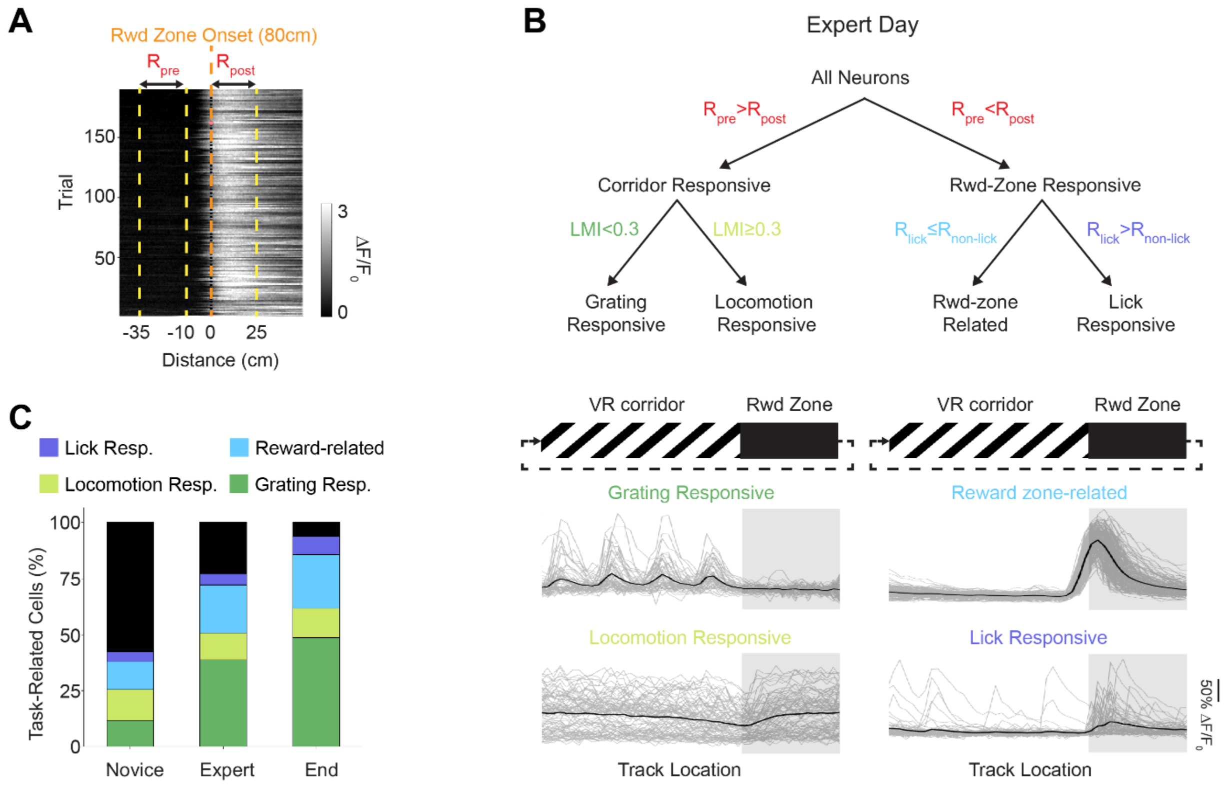

Figure S1. Response profile of V1 neurons during learning of a visually-guided reward task. Related to Figure 2.

(A) Normalised $\Delta F/F_0$ around the reward zone onset (orange dashed line) for a neuron defined as task-related (R_{pre} vs R_{post}; $p<0.001$, Wilcoxon signed rank test). The 25 cm block used to define activity before (R_{pre}) and after (R_{post}) the reward zone onset are marked for reference (yellow dashes lines).

(B) Flow chart defining the characterisation of different response types within the task-responsive V1 population. All task-responsive neurons were first separated into those significantly decreasing (corridor responsive; $R_{pre} > R_{post}$; $p<0.001$, Wilcoxon signed rank test) or increasing (reward zone responsive; $R_{pre} < R_{post}$; $p<0.001$, Wilcoxon signed rank test) their activity at the reward-zone onset. The corridor responsive neurons were sub-divided based on whether their locomotion modulation index (LMI) was < 0.3 (grating responsive) or > 0.3 (locomotion responsive). The reward-zone responsive neurons were sub-divided based on whether their activity during licking (R_{lick}) was significantly higher than their activity during an equal number of non-licking ($R_{non\text{-}lick}$) periods along the virtual corridor (lick responsive; R_{lick} vs $R_{non\text{-}lick}$; $p<0.001$, Wilcoxon signed rank test) or not (reward-zone related). Lower panels show $\Delta F/F_0$ for single trials (grey lines) and average response (black line) for representative neurons classified in each of the response types.

(C) Bar chart showing the evolution of the percentage of the different neuronal response types during learning. At each stage (novice, expert and end-point days) neurons were classified as described in B.

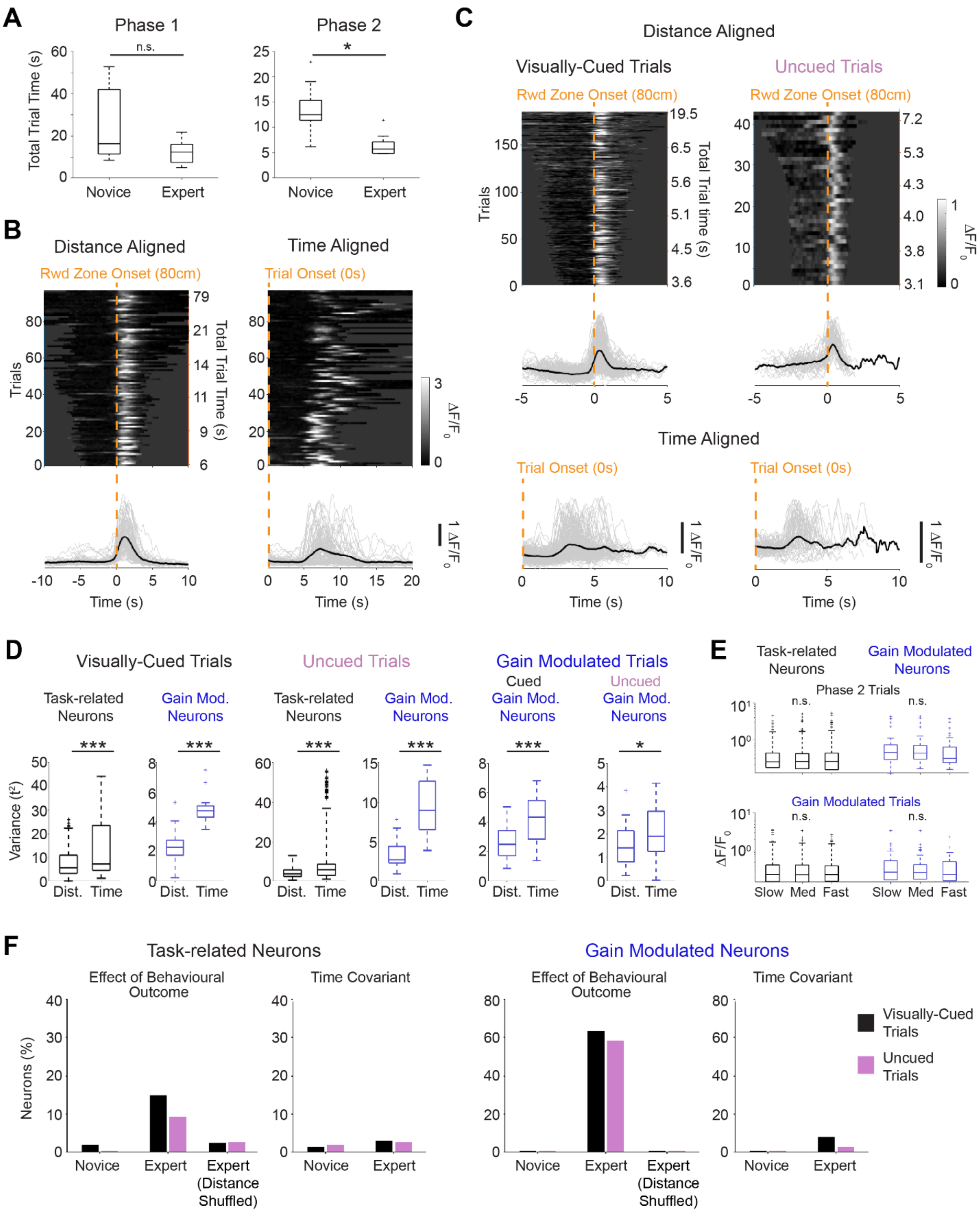

Figure S2. Contribution of trial time to task-related and gain-modulated neuronal responses. Related to Figure 2 and Figure 3.

(A) The average trial time per animal during novice and expert days is shown for both learning phases (Phase 1: p=0.219; Phase 2: p=0.016; n=7; Kruskal–Wallis test), showing that during the expert day animals completed trials faster and at a less variable rate. Boxplots represent median, 1st and 3rd quartile, and 1.5 interquartile range (IQR).

(B) The responses of an example neuron are shown (same neuron as shown in Figure S2B) for the expert day (Phase 1), over time and for each trial. Responses are aligned either to the reward-zone onset (at 80 cm; left panel) or to the start time of each trial (trial onset, 0 s; right). In the left panel, the trials are sorted by total trial time (indicated on the right y-axis). The bottom panels show the traces ($\Delta F/F_0$) of each trial (grey lines) and the mean across trials (black line). Note that this neuron responds reliably at the reward-zone onset (80 cm) for the fastest as well as slowest trials, which can differ by more than an order of magnitude (range 6-79 s). When responses are aligned to trial onset, the peak responses are more variable across trials (right panel).

(C) Example responses are shown from expert day for Phase 2 for visually-cued (left) and uncued (right) trials aligned to distance onset (top two panels; reward-zone onset, 80 cm) and trial times are sorted from fastest to slowest (indicated on the right y-axis). Note that even for uncued trials, there is a reliable response at the reward-zone onset (80 cm), regardless of whether the animal completes a trial quickly (3.1 s shortest trial time) or takes more than double that time (7.2 s). This indicates that the response to the uncued reward-location is not an entrainment response across trials of the same length but is specific to a spatially-defined reward location. In the bottom panels, $\Delta F/F_0$ traces for each trial (grey lines) and the mean across trials (black line) are shown aligned to the trial onset and show more variability.

(D) Trial-by-trial variability is quantified for all task-related neurons (black; see also Figure 2) and all gain-modulated neurons (blue; see also Figure 3E-F) on expert day Phase 2, by taking the variance of the time at peak response for each trial when trials were aligned either by distance (Dist.; reward-zone onset, 80 cm) or by trial start (Start; trial onset, 0 s). In both populations of neurons and for both visually-cued trials and uncued trials, as well as gain modulated trials (cued and uncued) we found significantly less variability when responses were aligned to distance onset (***$p<0.001$, *$p=0.0315$; task-related neurons $n = 613$; gain modulated neurons $n = 38$; Kruskal–Wallis test). Boxplots represent median, 1st and 3rd quartile, and 1.5 IQR.

(E) The mean $\Delta F/F_0$ surrounding the reward zone (10 cm before and after reward zone onset) is shown for the slowest trials (slow; 1st quartile), fastest trials (fast; 3rd quartile), and all trials in between (med) for the task-related (black) and gain modulated (blue) population of neurons. The top panel shows data from Phase 2 (non-gain modulated trials) on expert day and the bottom panel shows data from gain modulated trials (Phase 3). We found no significant difference between any condition ($p>0.050$ for all conditions, task-related neurons $n=613$; gain modulated neurons $n=38$; Kruskal–Wallis test). Boxplots represent median, 1st and 3rd quartile, and 1.5 IQR.

(F) The percentage of task-related (left) and gain modulated (right) neurons in V1 with significantly different responses according to behavioural outcome (successful vs missed trials) according to a multivariate analysis of covariance (MANCOVA) and taking into account total trial time as a covariate, for each of the different stimulus conditions (visually-cued [black] vs uncued [purple] trials). On expert days, the proportion of neurons showing responses affected by behavioural outcome was larger than on novice days (especially in gain modulated neurons). When the distance along the track was shuffled (so that random lengths of the track outside the reward-zone were sampled; distance shuffled), the effect of behavioural outcome was abolished. Note that time was not a significant covariate for the majority of neurons on either novice or expert days for either stimulus condition. For all conditions significance was determined by $p<0.001$.

SUPPLEMENTAL METHODS

Water deprivation and training schedule

Mice were put on a 1 ml/day water restriction regime to increase motivation during behavioral training. This regime maintained bodyweight at 85-90% of their free feeding weight, calculated as the mean of the last 3 days before water restriction. Where possible, mice were trained at the same time every day and generally in the first few hours of their dark phase. Training consisted of a 30 minute session, 5 days per week, followed by a 2-day break. Successful trials were rewarded with a droplet of water (~ 5μl/reward). Following each session, the volume of water consumed during the task was supplemented to 1ml if necessary.

Virtual Reality gain modulation

For phase 3 the gain relating the rotation of the cylindrical treadmill to the progression in the virtual corridor was reduced from 1 to 0.75. This resulted in a mismatch between the movement on the treadmill and movement through the virtual environment. On these trials, the same number of steps took animals only 75% of the distance along the virtual corridor compared to a normal trial (see Figure 3E). In these trials, the reward was given at 80 cm along the virtual corridor (in virtual space), which corresponds to 107 cm in physical distance travelled. Therefore, the reward was actually given after the expected reward onset based on physical distance along the treadmill (the expected location being at 80 cm of physical distance travelled and correspondingly 60 cm of virtual space along the virtual corridor; see also axes in Figure 3E, F). The gain modulated trials were all performed in one session with trials interleaved such that 1:5 was gain modulated and 1:10 was gain modulated and uncued.

Data analysis

The virtual reality system was updated at a rate of 60 Hz. At each frame, custom code was executed that updated the state of the virtual environment and triggered events (e.g. dispense a reward) when appropriate. At the end of each frame, one line was added to the behavioral raw data file containing the necessary information for post-hoc data analysis (time since start of session, location, occurrence of a lick, etc.). Spatial displacement of the mouse in the virtual environment was calculated as the mean treadmill rotation recorded over the preceding 200 ms. Imaging and behavioral datafiles were aligned post-hoc. The behavioral datafile was matched to the imaging datafile by downsampling and interpolating such that the aligned dataset had the same number of frames. Binary behavioral events that could not be interpolated, such as licking or reward, were matched to the closest frame of the imaging dataset.

For classifying task-related neurons, we compared the activity within 25 cm blocks taken before and after the reward-zone onset for each trial (Rpre vs Rpost; p<0.001, Wilcoxon signed rank test; see Figure S1A). If R_{pre} (-35 to -10 cm before the reward-zone onset) was significantly greater than R_{post} (0 to 25 cm after the reward-zone onset), then the neuron decreases its activity at the reward zone and was categorized as corridor responsive. If R_{post} was significantly greater than R_{pre}, then the neuron was considered reward-zone responsive (Figure S1).

We quantified the effect of locomotion on neuronal activity by using a locomotion modulation index (LMI), which is the difference between the ΔF/F0 during locomotion (R_L) and stationary (Rs) periods, normalized by the activity during both periods: LMI = (R_L - Rs) ⁄ (R_L + Rs) (see also Pakan et al., 2016).

To quantify the accuracy by which V1 activity could be classified based on the neuronal population activity, we used a template-matching decoder (Montijn et al., 2014), which compares the population activity per trial to response templates of the different experimental conditions (i.e. successful vs missed trials; visually-cued vs uncued trials). These templates are generated by taking the mean ΔF/F0 immediately prior to and within the reward zone (from 5 cm before the reward zone to the end of the reward zone) for each neuron in a single field of view, resulting in a template of population activity (Rt) per trial. The similarity of this template to the actual population activity (RP) for all other trials per condition is given by:

$$I_t = \frac{\sum_{i=1}^{N} R_i^P \cdot R_i^t}{|R^t| \cdot |R^P|},$$

where i indexes the N elements (neurons) of R. The similarity index I is calculated for all conditions and the decoded output is determined by taking the condition with the highest similarity to the template population activity. Decoder accuracy is given by the percentage of correctly decoded trials.

Multivariate analysis of covariance was performed for each stimulus condition (visually-cued and uncued trials) to test for the significance of a group effect of behavioral outcome (successful vs missed trials) with total trial time as a covariate for each neuron (see Figure S2).

Statistics

Error bars in all graphs indicate standard error of the mean (s.e.m.) and statistics were performed with two-tailed tests. Unless otherwise stated, for statistical tests comparing values across days or conditions (e.g. cued/uncued) for the same population of neurons, we used the Wilcoxon signed rank test (paired difference test). For statistical tests comparing measures across conditions that involved different underlying population of neurons, we used the Kruskal–Wallis test (one-way ANOVA on ranks). When analysing data from all neurons, we used the number of animals as our sample size because neuronal responses from the same mouse may be correlated and thus do not represent independent samples. Therefore, comparing measures across neurons, rather than across animals, may incorrectly inflate the degrees of freedom with the risk of false positive results for detecting significant differences.
